# Editorial: Recent Development of Nanocatalysts for Hydrogen Production

**DOI:** 10.3389/fchem.2020.00576

**Published:** 2020-07-09

**Authors:** Quanbing Liu, Lijun Yang, Sheng Chen, Bruno G. Pollet, Hao Li

**Affiliations:** ^1^School of Chemical Engineering and Light Industry, Guangdong University of Technology, Guangzhou, China; ^2^Ballard Power Systems, Burnaby, BC, Canada; ^3^School of Chemistry, University of New South Wales, Sydney, NSW, Australia; ^4^Hydrogen Energy and Sonochemistry Research Group, Department of Energy and Process Engineering, Faculty of Engineering, Norwegian University of Science and Technology (NTNU), Trondheim, Norway; ^5^School of Chemistry and Materials Engineering, Huizhou University, Huizhou, China

**Keywords:** hydrogen production, nanocatalysts, heterogeneous catalysis, electrocatalysis, photocatalysis

Hydrogen is a green and sustainable energy carrier that has been recognized as one of the most promising candidates to replace traditional fossil fuels in the future. Because of the absence of hydrogen in nature, the production of hydrogen by different artificial catalytic processes has received extensive attentions over the past decades. In these processes, nanocatalysts play critical roles in all aspects of the hydrogen generation. Further large-scale commercialization of these hydrogen production technologies is desirable, but there are several issues that should be addressed such as reducing the fabrication costs of nanocatalysts to an acceptable level, improving the catalytic activity and selectivity of the nanocatalysts, and enhancing the operation stability of the nanocatalyst electrodes. Therefore, the exploration of low-cost routes for preparing nanocatalysts, especially those noble-metal free catalysts that can be easily scaled up to industrial processes, is of extreme importance.

Heterogeneous catalysis, particularly the catalytic dehydrogenation of ammonia borane (AB), is a safe and effective pathway to produce hydrogen. Other than traditional noble-metal and first-row transition (Co, Ni, Cu) metallic catalysts, oxide-based compounds/composites have emerged as a new type of high-performance catalysts. For example, Cu_*x*_Co_1−x_O nanoparticles supported on graphene oxide show a high turnover frequency (TOF) of 70.0 mol_hydrogen_ min^−1^ mol_cat_.^−1^ in AB hydrolysis (Feng et al., 2016). Lu et al. have prepared a series of Cu_*x*_Ni_1−x_Co_2_O_4_ nanowires catalysts toward the hydrolysis of ammonia borane and achieve the largest turnover frequency (TOF) of 119.5 mol_hydrogen_ min^−1^ mol_cat_.^−1^ at *x* = 0.6 (Lu et al., 2018). Other successful example catalysts include bracelet-like Ni_0.4_Cu_0.6_O microstructure (Li et al.), Cu_0.5_Co_0.5_O nanocubes (Zheng et al., 2018), CuO-NiO/Co_3_O_4_ hybrid nanoplates (Liao et al., 2020). In these catalysts, significant synergistic effects between different oxides in these compound/composites have been observed i.e., the oxide-based compound/composites usually exhibit a higher catalytic activity than their corresponding metal or alloy components. The detailed mechanisms are still not well-understood, and further investigation is in progress. Other than AB, recent studies have shown the production of hydrogen by dehydrogenation of other feedstocks like formic acid, methanol, ethanol, etc. under mild conditions. For example, Ortega-Murcia et al. have synthesized PVP-capped Pd nanoparticles supported on various categories of carbon substrates for the dehydrogenation of formic acid. They have discovered that MWCNT-supported catalyst displayed the best performance, which was attributed to the characteristic 1D structure and highly available external surface area of MWCNTs (Ortega-Murcia et al.).

On the other hand, electrocatalytic hydrogen production is currently one of the best solutions to explore the conversion and lone-term storage of surplus electricity from renewable energy. According to different electrolytes, there is alkaline, neutral, and acidic electrocatalytic hydrogen production, where high-efficiency nanocatalysts are the most important elements in these systems. In addition to the traditional precious metal catalysts, many new emerging non-noble nanoalloys and non-metallic nano-carbon materials and their derivatives have exhibited excellent catalytic activity for hydrogen evolution reaction (HER). For example, Liu et al. have reported a two-step procedure combining of hydrothermal and high temperature heat-treatment to fabricate a hollow porous Mo_2_C@C nanospheres, which show an ultralow Tafel slope (~55 mV dec^−1^), small overpotential (~167 mV at 10 mA cm^−2^), and strong cycling durability in 0.5 M H_2_SO_4_ solution. The same group also has synthesized a non-precious-metal catalyst (CoO_*x*_) for HER through a facile hydrothermal process, which shows excellent catalytic activity (over-potential of 112 mV at 20 mA cm^−2^, Tafel slope of 94 mV dec^−1^) (Wu et al.). Moreover, Zeng et al. have prepared a series of differently shaped NiMnO catalysts, and highlight the important role of PEG additive in the formation of abundant petal-like nanostructures, which could lead to high specific surface area and consequently excellent HER activity in alkaline medium. Further, Djara et al. have successfully synthesized self-support Ni_3_S_2_ particles embedded into a nitrogen-sulfur-nickel-carbon nanostructure network for promoting HER in 1 M KOH alkaline medium. They found that the overpotential was only 194 mV (10 mA cm^−2^) and the Tafel slope was 84 mV dec^−1^ for HER after 24 h of continuous operation (Djara et al.). Considering the above successful examples, it is believed that the rational manipulation of microstructures and compositions of nanocatalysts is crucial for the development of high-performance hydrogen evolution electrode.

Recently, with the fast development of synthetic and characterization technology of nanocatalysts, significant progress has been witnessed in improving their photocatalytic efficiency and the understanding of basic principles of semiconductor photocatalysis. For photocatalytic hydrogen production, the semiconductor nanophotocatalyst is first exposed to light irradiation. When the energy of incident photons exceeds the band gap of semiconductors, electrons will be excited from valence to conduction band, which then meet with H^+^ in the reduction solution, and consequently generate hydrogen gas. For example, Peng et al. have prepared single-layer MoS_2_ nanosheets by ultrasonic-assisted peeling method, which showed a high hydrogen production at 60°C (Peng et al., 2016). Their work manifested the confluence of optical, electronic and chemical properties of 2D MoS_2_ monolayers that could be fully captured for efficient photocatalytic water reduction. Yang et al. have prepared a series of Ag_2_O-TiO_2_ hybrid nanoparticles with different morphologies. It was found that 20% Ag_2_O-TiO_2_ nanospheres mixed with 80% Ag_2_O-TiO_2_ nanoplates displayed the best photocatalytic activity, which can be attributed to the improved colloidal dispersion stability (Yang et al.). Although significant progress has been made, there are still big challenges in the field of photocatalysis. One can refer to Prof. Ohtani's paper, which includes insightful observations in this area (Ohtani, 2017).

In current theme issue, we have collected eight valuable contributions containing of preparation, microstructure control, and applications of various nanocatalysts for hydrogen production. We hope that these contributions might provide some new insights into the development of high-performance nanocatalysts, especially noble-metal-free nanocatalysts, for the hydrogen production. We are pleased to see booming publication in this area discussing the relationships between morphologies, structures, surface states, crystallinity, the defects of nanocatalysts and their hydrogen production activity/selectivity/stability, which will benefit to design novel catalysts with high performance.

## Author Contributions

All authors listed have made a substantial, direct and intellectual contribution to the work, and approved it for publication.

## Conflict of Interest

LY was employed by the Ballard Power Systems, Burnaby. The remaining authors declare that the research was conducted in the absence of any commercial or financial relationships that could be construed as a potential conflict of interest.
